# Correction: Tissue Expression Pattern of PMK-2 p38 MAPK Is Established by the miR-58 Family in *C*. *elegans*

**DOI:** 10.1371/journal.pgen.1005317

**Published:** 2015-07-30

**Authors:** The PLOS Genetics Staff

## Notice of Republication

This article was republished on May 26, 2015, to include details of the editor who handled the submission, which had been omitted due to an error in production. Please download this article again to view the correct version. The originally published, uncorrected article and the republished, corrected article are provided here for reference.

## Supporting Information

S1 File Originally published, uncorrected article. (PDF)

S2 File Republished corrected article. (PDF)

## References

[1] Pagano DJ, Kingston ER, Kim DH (2015) Tissue Expression Pattern of PMK-2 p38 MAPK Is Established by the miR-58 Family in *C*. *elegans* . PLoS Genet 11(2): e1004997 doi: 10.1371/journal.pgen.1004997 25671546 10.1371/journal.pgen.1004997 PMC4335502

