# Supplementary material for: Tissue Expression Pattern of PMK-2 p38 MAPK Is Established by the miR-58 Family in C. elegans
Source: PLoS Genet. 2015 Feb 11;11(2):e1004997. doi: 10.1371/journal.pgen.1004997 (PMC4335502; doi:10.1371/journal.pgen.1004997)

A

GAAGAGCCAATGAAAATATGAagtcaaatgtgttcaagtgctaaaat**atctca**aaactgtggaattttctttttttctatattgtatctccctaaactatttat
ttcactaaaaatcaaatttgtgtccctaattcct**tgatctct**aattatccaacaat**cgatctca**gatgttcttttccacaaa**atctca**atttttttcctttgaa
accatagacttgtgatttaccttaactttctcgttaatcctataagttgccatgacctcagagcctcttttaatttattattcatttagattcatttatcaaa
ttgaagtgtataaagaattaatgtaaatattcaattttctaaatactcttaaacatttaatcggttcaaattgccgcttaatttttatatttacatttatatt
ggttttacgtacacaaaaaaatcatattttttttcaaaataattacttcaaatcttacaaacatgattccaggtaacggagccaATGTTTCCACAGACAACAAT

B

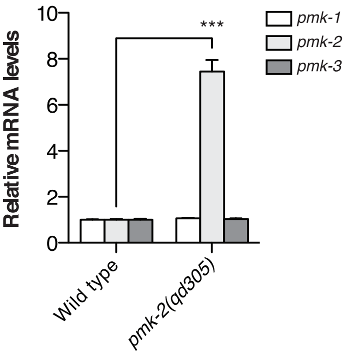

C

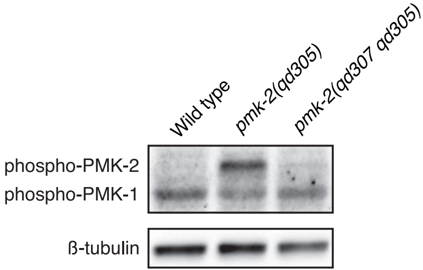

Supplement: S1 Fig — (A) DNA sequence of the pmk-2 3’UTR (release WS245). Uppercase, last exon of pmk-2 and first exon of pmk-1, respectively. Underline, polyadenylation signal. Bold font, miR-58/80-82 family seed match site. Gray highlight, qd305 deletion. (B) qRT-PCR analysis of pmk-1, pmk-2, and pmk-3 mRNA levels in L4 larval stage wild type worms and the pmk-2(qd305) mutant. Levels of pmk-1, pmk-2, and pmk-3 mRNA are normalized to the levels of snb-1 mRNA. Values plotted are the fold changes relative to wild type. Shown is the mean ± SEM (n = 3 independent biological replicates, *** P<0.001, two-way ANOVA with Bonferroni post-test). (C) Immunoblot analysis of lysates from L4 larval stage wild type worms, pmk-2(qd305) mutant animals, and pmk-2(qd307 qd305) intragenic suppressor mutant animals using antibodies that recognize activated p38 MAPK and β-tubulin. (PDF) [file pgen.1004997.s001.pdf]
