# Supplementary figures and images for: Tissue Expression Pattern of PMK-2 p38 MAPK Is Established by the miR-58 Family in C. elegans
Source: PLoS Genet. 2015 Feb 11;11(2):e1004997. doi: 10.1371/journal.pgen.1004997 (PMC4335502; doi:10.1371/journal.pgen.1004997)

Wild type

mir-80; mir-58; mir-81-82

mir-80; mir-58 pmk-2(qd284); mir-81-82

phospho-PMK-2  
phospho-PMK-1

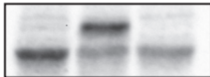

$\beta$ -tubulin

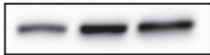

Supplement: S2 Fig — Immunoblot analysis of lysates from L4 larval stage wild type worms, mir-80; mir-58; mir-81-82 mutant animals, and mir-80; mir-58 pmk-2(qd284); mir-81-82 mutant animals using antibodies that recognize activated p38 MAPK and β-tubulin. (PDF) [file pgen.1004997.s002.pdf]
