# Supplementary material for: Tissue Expression Pattern of PMK-2 p38 MAPK Is Established by the miR-58 Family in C. elegans
Source: PLoS Genet. 2015 Feb 11;11(2):e1004997. doi: 10.1371/journal.pgen.1004997 (PMC4335502; doi:10.1371/journal.pgen.1004997)
Supplement: S1 Table — (PDF) [file pgen.1004997.s003.pdf]

Strain	Genotype
AU78	*agIs219 III*
ZD395	*agIs219 III; sek-1(km4) X*
ZD39	*agIs219 III; pmk-1(km25) IV*
ZD1050	*agIs219 III; pmk-2(qd284) IV*
ZD1053	*agIs219 III; pmk-2(qd287) IV*
ZD983	*agIs219 III; pmk-2(qd279 qd171) pmk-1(km25) IV*
ZD1080	*agIs219 III; pmk-2(qd280 qd171) pmk-1(km25) IV*
ZD832	*kyIs140 I*
ZD896	*kyIs140 I; sek-1(km4) X*
ZD881	*kyIs140 I; pmk-1(km25) IV*
ZD1055	*kyIs140 I; pmk-2(qd284) IV*
ZD1054	*kyIs140 I; pmk-2(qd287) IV*
ZD949	*kyIs140 I; pmk-2(qd279 qd171) pmk-1(km25) IV*
ZD1007	*kyIs140 I; pmk-2(qd280 qd171) pmk-1(km25) IV*
ZD827	*nIs145 X*
ZD1079	*tir-1(qd4) III; nIs145 X*
ZD874	*pmk-1(km25) IV; nIs145 X*
ZD1051	*pmk-2(qd284) IV; nIs145 X*
ZD1056	*pmk-2(qd287) IV; nIs145 X*
ZD985	*pmk-2(qd279 qd171) pmk-1(km25) IV; nIs145 X*
ZD1057	*pmk-2(qd280 qd171) pmk-1(km25) IV; nIs145 X*
ZD1195	*qdEx101[P_{operon}::islo-1::pmk-3::pmk-2::GFP::pmk-1::mCherry]*
ZD721	*agIs219 III; pmk-2(qd171) pmk-1(km25) IV*
N2	wild type
ZD1221	*pmk-2(qd305) IV*
ZD1226	*pmk-2(qd307 qd305) IV*
MT17453	*mir-58(n4640) IV*
MT13949	*mir-80(nDf53) III*
MT13954	*mir-81-82(nDf54) X*
MT18409	*mir-80(nDf53) III; mir-58(n4640) IV*
MT18410	*mir-58(n4640) IV; mir-81-82(nDf54) X*
MT14128	*mir-80(nDf53) III; mir-81-82(nDf54) X*
MT15563	*mir-80(nDf53) III; mir-58(n4640) IV; mir-81-82(nDf54) X*
ZD1196	*qdEx102[P_{operon}::islo-1::pmk-3::pmk-2_{mut}::GFP::pmk-1::mCherry]*
ZD1199	*mir-80(nDf53) III; mir-58(n4640) IV; mir-81-82(nDf54) X; qdEx101[P_{operon}::islo-1::pmk-3::pmk-2::GFP::pmk-1::mCherry]*
ZD1205	*mir-80(nDf53) III; mir-58(n4640) pmk-2(qd284) IV; mir-81-82(nDf54) X*
