# Supplementary material for: Correction: Tissue Expression Pattern of PMK-2 p38 MAPK Is Established by the miR-58 Family in C. elegans
Source: PLoS Genet. 2015 Jul 30;11(7):e1005317. doi: 10.1371/journal.pgen.1005317 (PMC4520587; doi:10.1371/journal.pgen.1005317)
Supplement: S1 File — (PDF) [file pgen.1005317.s001.pdf]

PLOS | GENETICS

RESEARCH ARTICLE

Tissue Expression Pattern of PMK-2 p38 MAPK Is Established by the miR-58 Family in *C. elegans*

Daniel J. Pagano, Elena R. Kingston, Dennis H. Kim*

Department of Biology, Massachusetts Institute of Technology, Cambridge, Massachusetts, United States of America

* dhkim@mit.edu

Abstract

Analyses of gene expression profiles in evolutionarily diverse organisms have revealed a role for microRNAs in tuning tissue-specific gene expression. Here, we show that the relatively abundant and constitutively expressed miR-58 family of microRNAs sharply defines the tissue-specific expression of the broadly transcribed gene encoding PMK-2 p38 MAPK in *Caenorhabditis elegans*. Whereas PMK-2 functions redundantly with PMK-1 in the nervous system to regulate neuronal development and behavioral responses to pathogenic bacteria, the miR-58, miR-80, miR-81, and miR-82 microRNAs function redundantly to destabilize *pmk-2* mRNA in non-neuronal cells with switch-like potency. Our data suggest a role for the miR-58 family in the establishment of neuronal-specific gene expression in *C. elegans*, and support a more general role for microRNAs in the establishment of tissue-specific gene expression.

CrossMark
click for updates

OPEN ACCESS

Citation: Pagano DJ, Kingston ER, Kim DH (2015) Tissue Expression Pattern of PMK-2 p38 MAPK Is Established by the miR-58 Family in *C. elegans*. PLoS Genet 10(2): e1004997. doi:10.1371/journal.pgen.1004997

Received: December 10, 2014

Accepted: January 12, 2015

Published: February 11, 2015

Copyright: © 2015 Pagano et al. This is an open access article distributed under the terms of the Creative Commons Attribution License, which permits unrestricted use, distribution, and reproduction in any medium, provided the original author and source are credited.

Data Availability Statement: All relevant data are within the paper and its Supporting Information files.

Funding: This work was supported by NIH Grant R01-GM084477. The funders had no role in study design, data collection and analysis, decision to publish, or preparation of the manuscript.

Competing Interests: The authors have declared that no competing interests exist.

Author Summary

MicroRNAs are small, noncoding RNAs that act post-transcriptionally to inhibit expression of their target mRNAs. Gene expression studies of microRNAs and their target transcripts in diverse organisms have suggested that microRNAs may function to shape patterns of tissue expression. In this paper, we show that the miR-58/80-82 family of microRNAs, which accounts for roughly half of all *C. elegans* microRNAs at all developmental stages, defines the spatial expression pattern of PMK-2 p38 MAPK. While the *pmk-2* gene is broadly transcribed, its tissue-specific expression is established by the redundant activities of miR-58, miR-80, miR-81, and miR-82, which switch off expression of PMK-2 through destabilization of *pmk-2* mRNA in non-neuronal tissues. Our data suggest a housekeeping role for the miR-58/80-82 family in establishing and maintaining neuronal patterns of gene expression in *C. elegans*, and supports a more general role for microRNAs in establishing patterns of tissue expression.

Introduction

Since the initial genetic identification of microRNAs in *Caenorhabditis elegans* [1,2], biochemical cloning methods and computational approaches have identified hundreds of microRNAs [3,4], though genetic analysis has defined functional roles for relatively few of these [5,6]. A single microRNA, miR-58, constitutes nearly half of all microRNAs in *C. elegans*, with constitutive expression in non-neuronal tissues through all developmental stages [7,8]. Differential RNA binding protein (RBP) immunoprecipitation with subsequent mRNA and protein quantification analyses (RIP-chip-SRM) has indicated the presence of hundreds of miR-58 targets [9]. Whereas deletion of miR-58 does not cause any apparent defects, a strain carrying deletions in the miR-58 family, comprised of miR-58 and the homologous microRNAs miR-80, miR-81, and miR-82, exhibits multiple mutant phenotypes, including defects in size, locomotion, and reproductive egg-laying [6].

Three *C. elegans* genes with homology to mammalian p38 MAPK—*pmk-1*, *pmk-2*, and *pmk-3*—are in a single operon along with an additional upstream gene, *islo-1* (Fig. 1A). PMK-1 and PMK-2 are highly homologous, sharing a 62% amino acid sequence identity and have the signature TGY motif found in the activation loop of p38 MAPKs [10]. PMK-1 regulates innate immunity in the intestine of *C. elegans* and is activated by a MAPK signaling cassette composed of p38 MAPK kinase SEK-1 and the MAPKKK NSY-1, homologous to mammalian MKK3/6 and ASK1, respectively [11,12]. Functioning upstream of NSY-1 and required for activation of PMK-1 in *C. elegans* is TIR-1, a conserved Toll-Interleukin-1 Receptor domain adaptor protein orthologous to mammalian SARM [13,14]. TIR-1-NSY-1-SEK-1 functions in the nervous system to regulate the specification of neuronal asymmetry in the AWC neuron pair [15–17], reproductive egg-laying behavior, and the upregulation of serotonin biosynthesis in the ADF chemosensory neurons in response to infection by *Pseudomonas aeruginosa* [12], but the MAPK targeted in the nervous system for these processes has not been defined, with *pmk-1* loss-of-function not affecting these neuronal phenotypes.

Here, we show that PMK-2 functions redundantly with PMK-1 in the nervous system of *C. elegans* to regulate development and behavioral responses to pathogenic bacteria, whereas PMK-1 alone functions in the intestine to regulate innate immunity. We observe distinct tissue expression patterns for the co-operonic *pmk-1* and *pmk-2* genes; in contrast to the ubiquitous expression pattern of PMK-1, PMK-2 is largely restricted to the nervous system. Tissue-specific expression of PMK-2 is established by the miR-58 family, which switches off expression of PMK-2 in non-neuronal tissues. Our data suggest a role for the relatively abundant miR-58 microRNA in the establishment of tissue-specific gene expression in *C. elegans*.

Results

Tissue-specific activities of p38 MAPK genes in *C. elegans*

We used mutant alleles of *pmk-1* and *pmk-2* to confirm that PMK-1 alone is required for expression of an intestinal reporter for p38 MAPK activity and innate immunity to infection by *P. aeruginosa* in the intestine. The *agIs219* reporter transgene contains the green fluorescent protein (GFP) gene fused to the promoter of the PMK-1-regulated gene *T24B8.5*, which is predicted to encode a peptide homologous to ShK toxin peptides, and serves as an *in vivo* readout of p38 MAPK activity in the intestine [12]. Expression of *agIs219* in the intestine is extremely diminished in the *pmk-1(km25)* mutant (Fig. 1B). In contrast, expression of *agIs219* is unchanged in *pmk-2(qd284)* and *pmk-2(qd287)* mutant animals (Fig. 1B). Similarly, in contrast to the enhanced susceptibility to *P. aeruginosa* observed in *pmk-1(km25)* mutant animals, *pmk-2*

A

B

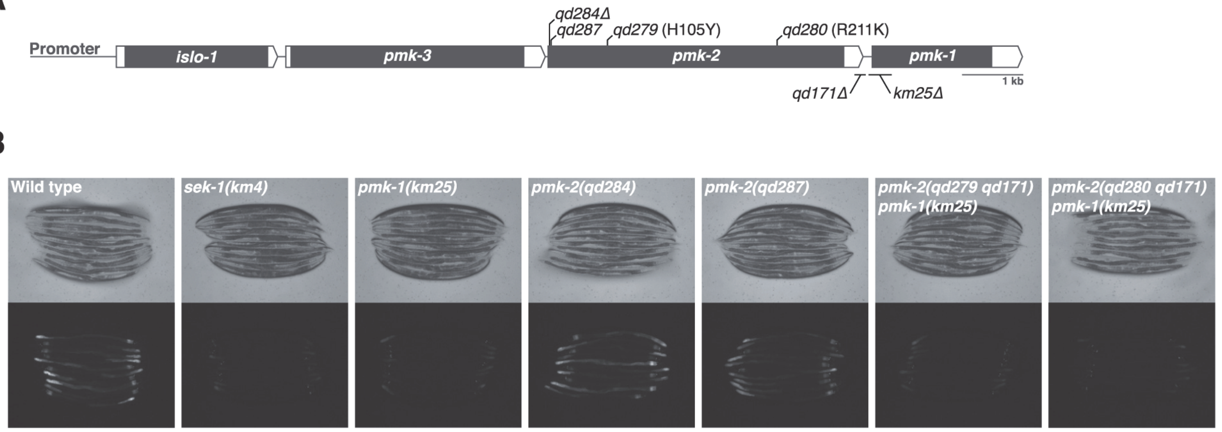

C

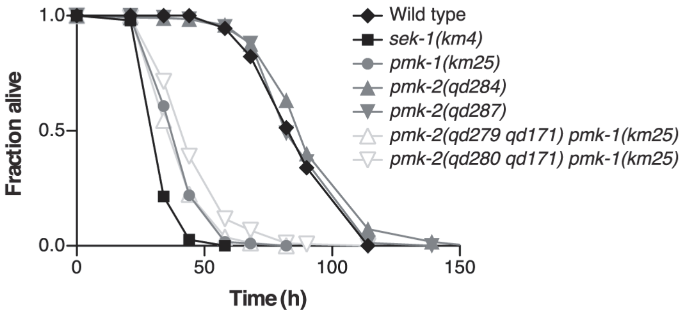

E

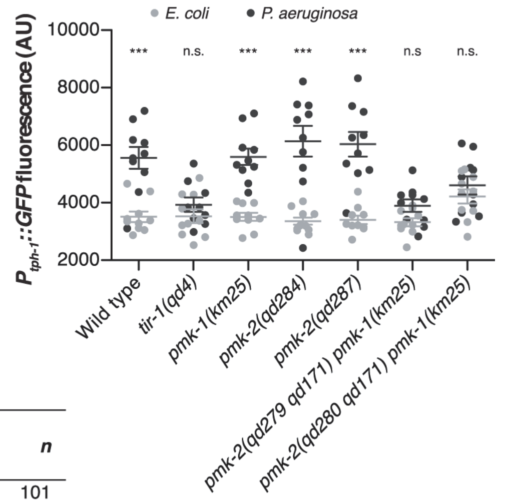

D

Genotype	Percentage of animals			*n*
	2 AWC^{OFF}	1 AWC^{ON} 1 AWC^{OFF}	2 AWC^{ON}	
Wild type	2	98	0	101
sek-1(km4)	0	0	100	95
pmk-1(km25)	1	97	2	103
pmk-2(qd284)	2	98	0	116
pmk-2(qd287)	1	99	0	102
pmk-2(qd279 qd171) pmk-1(km25)	1	3	96	108
pmk-2(qd280 qd171) pmk-1(km25)	0	7	93	101

F

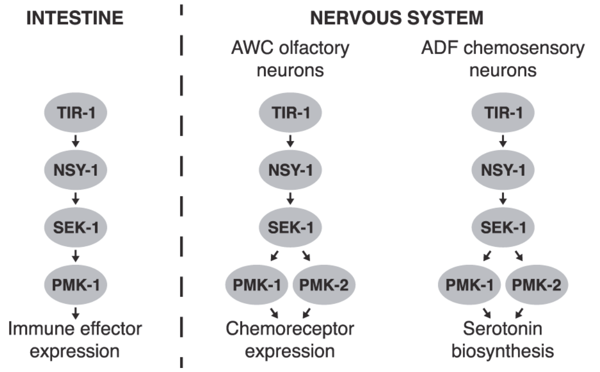

Fig 1. PMK-1 and PMK-2 function redundantly in the nervous system but not the intestine. (A) The *pmk* operon showing mutations utilized and isolated in this study. Gray fill, corresponding unspliced transcript; white fill, corresponding 5' and 3'UTRs. *pmk-2* mutations: *qd284*, 10 bp deletion, frameshift; *qd287*, 7 bp insertion, frameshift; *qd279* and *qd280*, as indicated in reference to isoform *pmk-2b* (release WS245); *qd171*, 913/184 bp insertion/deletion. *pmk-1*

mutation: *km25*, 375 bp deletion. (B-E) Phenotypic analysis of mutants deficient in p38 MAPK signaling. (B) Bright field and fluorescence microscopy images of 1-day-old adult worms carrying the *agIs219[P$_{T24B8.5}$::GFP]* transgene. (C) Pathogenesis assay of L4 larval stage worms on *P. aeruginosa* PA14. All strains carry the *agIs219[P$_{T24B8.5}$::GFP]* transgene. (D) Expression of *str-2::GFP* in the AWC olfactory neurons of L3 and L4 larval stage and young adult worms. (E) Quantification of GFP expression from the *nIs145[P$_{tph-1}$::GFP]* transgene in 1-day-old adult worms after a 6 hr exposure to *E. coli* OP50 or *P. aeruginosa* PA14. Shown is a representative experiment. Error bars, ± standard deviation. (n.s. not significant, *** $P<0.001$, two-way ANOVA with Bonferroni post-test). (F) PMK-1 p38 MAPK functions independently of PMK-2 p38 MAPK in the intestine downstream of the TIR-1-NSY-1-SEK-1 signaling module in the regulation of immune effector gene expression in response to pathogenic microbes. PMK-1 and PMK-2 p38 MAPKs function redundantly in the nervous system downstream of TIR-1-NSY-1-SEK-1 in the regulation of AWC neural asymmetry and pathogen-induced upregulation of *tph-1* expression in the ADF neurons.

doi:10.1371/journal.pgen.1004997.g001

(qd284) and *pmk-2(qd287)* mutant animals display a normal innate immune response to *P. aeruginosa* (Fig. 1C).

To evaluate the roles of PMK-1 and PMK-2 in mediating the activities of the TIR-1-NSY-1-SEK-1 signaling module in the nervous system, we utilized two assays of neuronal signaling processes that are dependent on TIR-1-NSY-1-SEK-1. First, the establishment of asymmetry in the AWC neurons during development is a stochastic process for which expression of a transgenic reporter, *str-2::GFP*, in one AWC neuron or the other serves as a readout [17]. TIR-1-NSY-1-SEK-1 signaling represses the expression of *str-2::GFP* in one AWC neuron such that in a wild type animal, only one AWC neuron expresses *str-2::GFP*. Strains carrying loss-of-function mutations in the TIR-1-NSY-1-SEK-1 signaling module express *str-2::GFP* in both AWC neurons [15–17]. We observed expression of *str-2::GFP* in only one AWC neuron in *pmk-1* and *pmk-2* loss-of-function single mutants (Fig. 1D). In contrast, using two strains carrying loss-of-function mutations in both genes—*pmk-2(qd279 qd171) pmk-1(km25)* and *pmk-2 (qd280 qd171) pmk-1(km25)*—we observed expression of *str-2::GFP* in both AWC neurons, similar to what is observed in the *sek-1(km4)* mutant (Fig. 1D). A second process that is dependent on TIR-1-NSY-1-SEK-1 activity in the nervous system is the increased expression of *tph-1*, which encodes the serotonin biosynthetic enzyme tryptophan hydroxylase, in the ADF chemosensory neurons upon exposure of *C. elegans* to pathogenic *P. aeruginosa* [12,18]. Upregulation of serotonin levels in the ADF neuron pair has been implicated in aversive learning behavior to pathogenic bacteria [18]. We observed that PMK-1 and PMK-2 also function redundantly in the *P. aeruginosa*-induced expression of a *P$_{tph-1}$::GFP* transgene reporter in the ADF neurons (Fig. 1E). These data establish that while PMK-1 function alone is required to regulate intestinal innate immunity, PMK-1 and PMK-2 function redundantly in the nervous system downstream of the TIR-1-NSY-SEK-1 signaling module in neuronal developmental and pathogen-dependent responses (Fig. 1F).

We reasoned that the genetic redundancy of *pmk-1* and *pmk-2* in neurons, but not in the intestine, might be the result of differences in tissue expression of these genes. We proceeded to examine the tissue expression patterns for PMK-1 and PMK-2 by constructing a translational reporter for the *pmk* operon, *qdEx101*, consisting of upstream promoter sequence and the entire length of the operon with a GFP tag engineered onto the C-terminus of PMK-2 and a mCherry tag engineered onto the C-terminus of PMK-1 (Fig. 2A). We observed broad expression of PMK-1 in multiple tissue types, whereas expression of PMK-2 was mostly restricted to the nervous system; PMK-2 expression was detected in head, body, and tail ganglia as well as the nerve ring and ventral nerve cord (Fig. 2B). We also observed faint and diffuse expression of PMK-2 in the distal tip cell and spermatheca at levels much lower than observed in the nervous system. Importantly, PMK-2 expression was excluded from the intestine, where PMK-1 functions solely in innate immunity. These data suggest that the tissue-specific genetic redundancy of p38 MAPK signaling in *C. elegans* is a consequence of distinct tissue expression patterns of the co-transcribed *pmk-1* and *pmk-2* genes and implicate post-transcriptional mechanisms in the tissue-specific regulation of *pmk-2* expression.

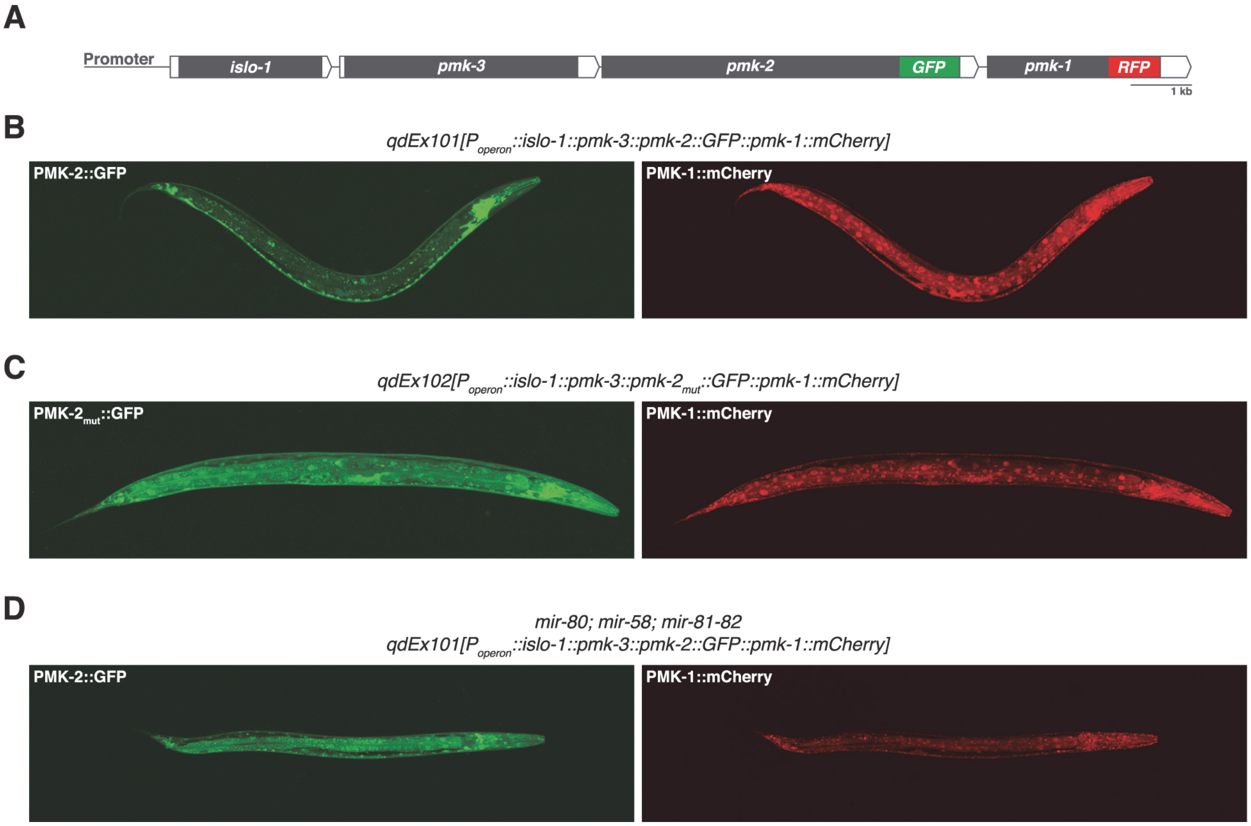

Fig 2. Distinct tissue expression patterns of *pmk-1* and *pmk-2*. (A) The *pmk* operon translational reporter. Green fluorescent protein was engineered onto the C-terminal end of PMK-2. The red fluorescent protein mCherry was engineered onto the C-terminal end of PMK-1. (B-D) Confocal fluorescence microscopy of a representative wild type worm carrying the *pmk* operon translational reporter (B), a wild type worm carrying a mutated *pmk* operon translational reporter with specific mutations (indicated in Fig. 4A) engineered into the second and third miR-58 family seed match sites in the 3'UTR of *pmk-2* (C), and a *mir-80; mir-58; mir-81-82* mutant worm carrying the *pmk* operon translational reporter (D).

doi:10.1371/journal.pgen.1004997.g002

The 3'UTR of *pmk-2* regulates mRNA stability

Insight into the post-transcriptional regulatory mechanism underlying the restricted tissue expression of PMK-2 came from a genetic screen for suppressors of the immunocompromised phenotype of a *pmk-1* deletion mutant [19], in which we serendipitously isolated a gain-of-function mutant of *pmk-2*, *qd171*, containing a 913 bp insertion/184 bp deletion located in the 3'UTR of *pmk-2* (Fig. 1A). The starting strain for the screen carried the *km25* deletion allele of *pmk-1* (Fig. 1A) and the *agIs219* integrated GFP reporter transgene [12,19] (Fig. 3A). The *pmk-2(qd171)* deletion suppressed the diminished intestinal GFP expression from the *agIs219* reporter transgene (Fig. 3A) and the enhanced pathogen susceptibility of the *pmk-1(km25)* mutant (Fig. 3B). RNAi of *sek-1* and RNAi of *pmk-2*, reverted the *pmk-2(qd171) pmk-1(km25)* pathogen resistance and *agIs219* intestinal GFP expression phenotypes associated with suppression of *pmk-1* loss-of-function (Fig. 3A-3B). These data suggest that the ability of the *pmk-2(qd171)* mutation to suppress *pmk-1* loss-of-function is dependent on PMK-2.

Because the location of the *qd171* insertion/deletion in the 3'UTR of *pmk-2* might be anticipated to influence mRNA stability, we proceeded to measure levels of *pmk-2* mRNA in the *pmk-2(qd171) pmk-1(km25)* mutant. We detected a 5.7-fold increase in *pmk-2* mRNA levels in the *pmk-2(qd171) pmk-1(km25)* mutant compared to wild type worms (Fig. 3C). The effect of increased mRNA levels due to the *qd171* mutation is specific to *pmk-2* and not a general

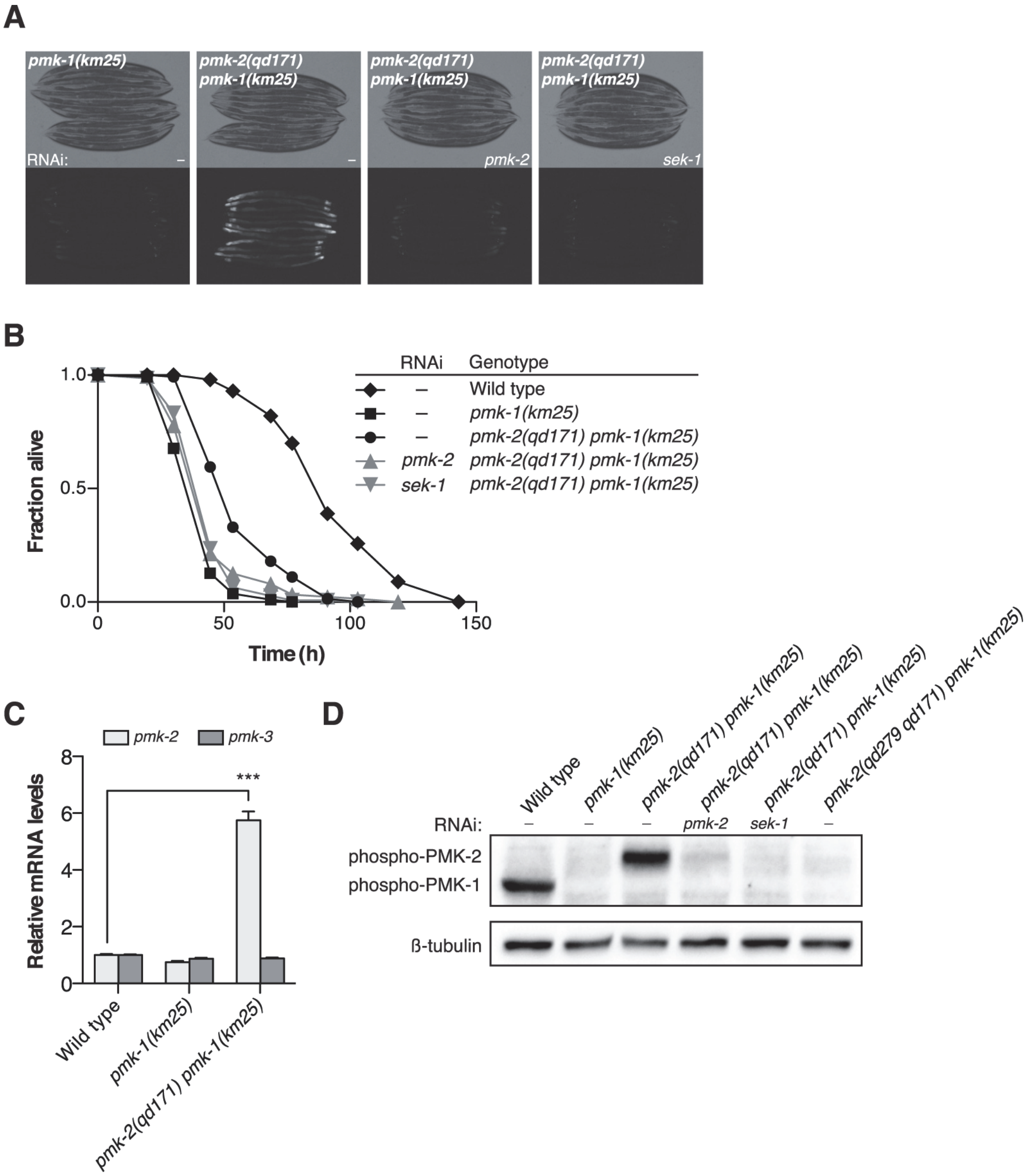

Fig 3. A deletion in the 3'UTR of *pmk-2* confers an increase in PMK-2 expression that can substitute for PMK-1 activity in the intestine. (A-B) Phenotypic analysis of the *pmk-2(qd171) pmk-1(km25)* mutant. Bright field and fluorescence microscopy images (A) and *P. aeruginosa* pathogenesis assays (B) of worms treated with RNAi as indicated for two generations. (C) qRT-PCR analysis of *pmk-2* and *pmk-3* mRNA levels in L4 larval stage animals. Levels of *pmk-2* and *pmk-3* mRNA are normalized to the levels of *snb-1* mRNA. Values plotted are the fold changes relative to wild type. Shown is the mean ± SEM (n = 4 independent biological replicates, *** $P<0.001$, two-way ANOVA with Bonferroni post-test). (D) Immunoblot analysis of lysates from RNAi-treated mixed stage animals using an antibody recognizing the doubly phosphorylated TGY motif of activated PMK-1 and PMK-2 p38 MAPKs and an antibody that recognizes β-tubulin. (A-D) All strains carry the *agIs219[$P_{T24B8.5}$::GFP]* transgene.

doi:10.1371/journal.pgen.1004997.g003

characteristic of the *pmk* operon, as *pmk-3* mRNA levels did not change in the *pmk-2(qd171) pmk-1(km25)* mutant relative to wild type. We next determined levels of activated PMK-2 protein in the *pmk-2(qd171) pmk-1(km25)* mutant using an antibody that recognizes the dually phosphorylated TGY motif in the activation domain of activated mammalian p38 MAPK and that is cross-reactive with *C. elegans* PMK-1 and PMK-2 [11]. Corroborating the increase in mRNA levels, we detected at least a 5.7-fold increase in activated PMK-2 protein levels in the *pmk-2(qd171) pmk-1(km25)* mutant (Fig. 3D).

To verify that the increase in levels of *pmk-2* mRNA and activated protein was due to the 3'UTR deletion of *pmk-2* in the *pmk-2(qd171) pmk-1(km25)* mutant, we used CRISPR-Cas-9-mediated genome editing to engineer the wild type N2 strain to carry a deletion in the 3'UTR of *pmk-2*. We obtained a 144 bp deletion, *qd305*, in the 3'UTR of *pmk-2* (S1A Fig.). We proceeded to measure levels of *pmk-2* mRNA and activated protein in the *pmk-2(qd305)* mutant and detected a 7.4-fold increase in *pmk-2* mRNA levels compared to wild type worms (S1B Fig.) and a corresponding increase in activated PMK-2 protein levels (S1C Fig.).

Taken together, these data suggest that a deletion in the 3'UTR of the gene encoding PMK-2 p38 MAPK can suppress the immunodeficient phenotype conferred by loss-of-function of *pmk-1* through increased stability of *pmk-2* mRNA and levels of activated PMK-2 protein.

The miR-58 family restricts expression of PMK-2 in *C. elegans*

We next sought to define the cis-regulatory determinants of the *pmk-2* 3'UTR that function to repress PMK-2 expression. The region deleted in the *qd171* allele of *pmk-2* contains the most distal polyadenylation signal (PAS) used in 3'-end formation of *pmk-2* mRNA. We performed 3' RACE on *pmk-2* mRNA isolated from wild type and *pmk-2(qd171) pmk-1(km25)* mutant animals to determine the effect of the *qd171* mutation on the length of the 3'UTR of *pmk-2* mRNA. Sequencing of the 3' RACE products revealed use of a more proximal PAS leading to a 206 bp truncation in the 3'UTR of *pmk-2* mRNA in the *pmk-2(qd171) pmk-1(km25)* mutant (Fig. 4A). We examined the truncated region for cis-regulatory elements conserved among *Caenorhabditis* species and identified three seed match sites for the miR-58/80-82 family of microRNAs residing in this region absent from the 3'UTR of *pmk-2* mRNA in the *pmk-2 (qd171) pmk-1(km25)* mutant (Fig. 4A). These miR-58/80-82 seed match sites are also absent in the *pmk-2(qd305)* mutant (S1A Fig.).

The miR-58/80-82 family consists of miR-58, miR-80, miR-81, miR-82, and miR-1834. Mutants carrying deletions in *mir-58*, *mir-80*, and *mir-81-82* [5] were used to assess whether the miR-58/80-82 family functions to repress the expression of *pmk-2*. Loss of any individual *mir-58/80-82* family member had no effect on *pmk*-2 mRNA levels relative to wild type (Fig. 4B). However, loss of both *mir-58* and *mir-80* resulted in a 3-fold increase in *pmk-2* mRNA levels relative to wild type, and loss of *mir-58/80-82* led to an even further increase in *pmk-2* mRNA to a level 6.3-fold greater than wild type (Fig. 4B), without altering levels of *pmk-3* mRNA. Corroborating the mRNA analysis, we observed at least similar increases in activated PMK-2 protein levels in both *mir-80; mir-58* and *mir-80; mir-58; mir-81-82* mutants, but not in other mutants (Fig. 4C). These data suggest that the miR-58/80-82 family acts redundantly to destabilize *pmk-2* mRNA.

The observation that the *pmk-2(qd171)* mutation suppresses the loss of *pmk-1* function in the intestine suggested that not only are PMK-2 levels increased in the *pmk-2(qd171) pmk-1 (km25)* mutant, but that the site of expression had also changed. To determine the spatial effect on expression of releasing *pmk-2* from regulation by the miR-58/80-82 family, we engineered a new *pmk* operon translational reporter, *qdEx102*, which carries mutations in the second and third miR-58/80-82 seed match sites [20] in the 3'UTR of *pmk-2* (Fig. 4A). Expression of PMK-1 was unchanged, whereas expression of PMK-2 was detected in many additional cell types including the intestine, body wall muscle, pharyngeal muscle, hypodermis, and vulva (Fig. 2C). Expression of PMK-2 in the distal tip cell and spermatheca, which was faint and diffuse with miR-58/80-82 regulation intact (Fig. 2B), was markedly elevated (Fig. 2C). Similar misexpression of *pmk-2* was observed when we crossed *qdEx101* (*pmk* operon reporter with miR-58/80-82 seed match sites intact) into the *mir-80; mir-58; mir-81-82* mutant (Fig. 2D). These data suggest that the miR-58/80-82 family of microRNAs restricts expression of PMK-2

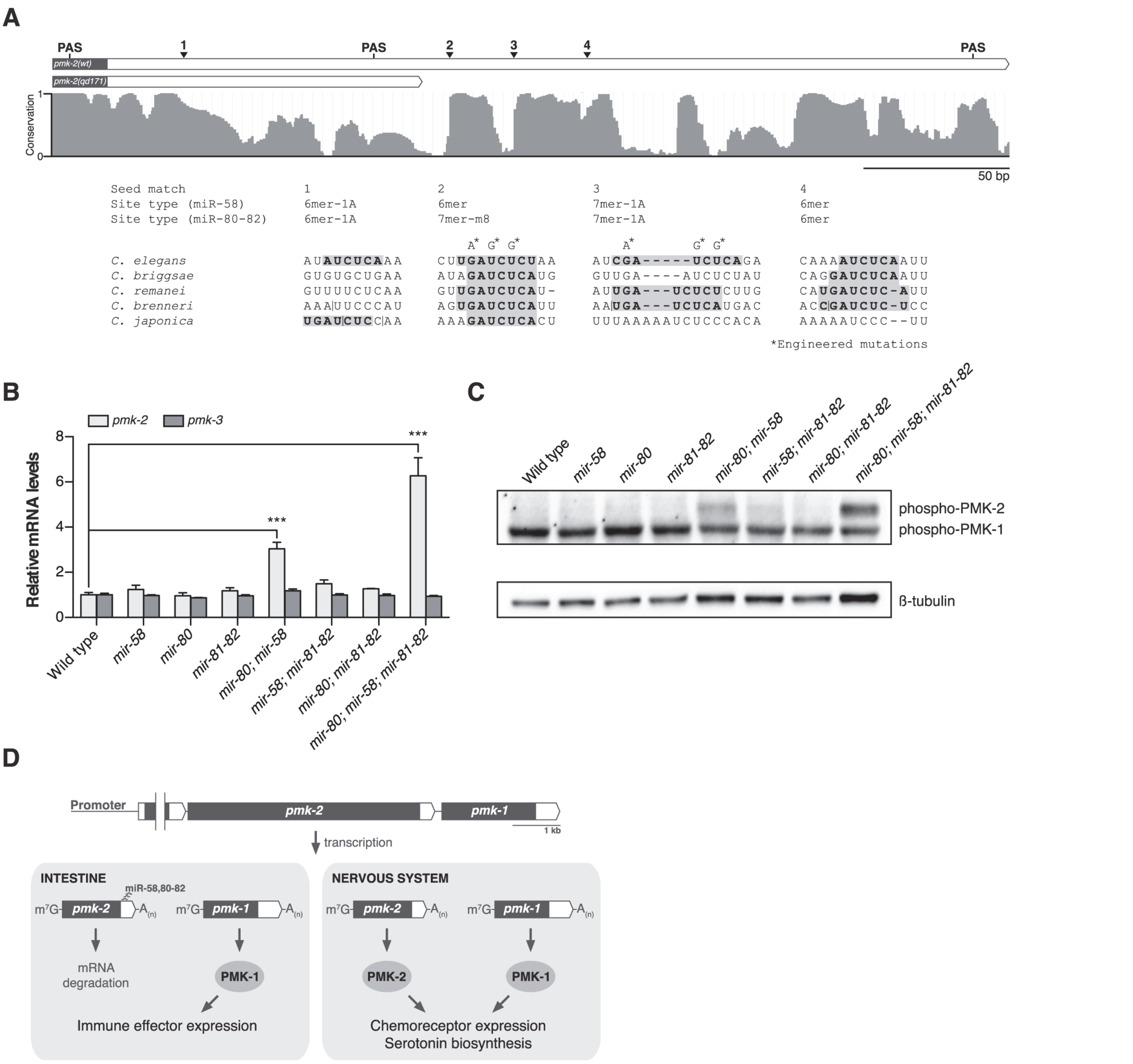

Fig 4. The miR-58/80-82 family of microRNAs functions redundantly to restrict expression of PMK-2 to the nervous system. (A) A schematic of the 3'UTR of *pmk-2* mRNA in wild type and *pmk-2(qd171) pmk-1(km25)* mutant animals as determined by 3' RACE analysis. Gray fill, exon; white fill, 3'UTR. Conservation of sequence among *Caenorhabditis* species by phastCons in shown [46]. miR-58/80-82 seed match sites are annotated. Vertical line, gap in alignment > 5 bp. Mutations engineered into the *pmk* operon translational reporter (Fig. 2C) are indicated. PAS, polyadenylation signal. (B) qRT-PCR analysis of *pmk-2* and *pmk-3* mRNA levels in L4 larval stage wild type worms and *mir-58/80-82* family mutants. Levels of *pmk-2* and *pmk-3* mRNA are normalized to the levels of *snb-1* mRNA. Values plotted are the fold changes relative to wild type. Shown is the mean ± SEM ($n = 3$ independent biological replicates, *** $P<0.001$, two-way ANOVA with Bonferroni post-test). (C) Immunoblot analysis of lysates from L4 larval stage wild type worms and *mir-58/80-82* family mutants using antibodies that recognize activated p38 MAPK and β-tubulin. (D) A model for the function of the miR-58/80-82 family of microRNAs in defining the tissue-specific expression of PMK-2 p38 MAPK through the post-transcriptional destabilization of *pmk-2* mRNA.

doi:10.1371/journal.pgen.1004997.g004

p38 MAPK through the post-transcriptional destabilization of *pmk-2* mRNA in non-neuronal tissues (Fig. 4D).

Discussion

Our data on the tissue-specific genetic redundancy of p38 MAPK signaling in *C. elegans* define a role for the relatively abundant and constitutively expressed miR-58/80-82 family of microRNAs in establishing the tissue-specific expression of PMK-2 p38 MAPK. We show that although the *pmk-2* gene is widely transcribed, PMK-2 protein is found nearly exclusively in the nervous system of *C. elegans* where PMK-2 functions redundantly with PMK-1 to regulate neuronal development and behavioral responses to pathogenic bacteria. We determined that the tissue-specific expression of PMK-2 is dependent on cis-regulatory sequences found within its 3'UTR and demonstrated that the miR-58/80-82 family is required to switch off expression of PMK-2 in non-neuronal tissues post-transcriptionally, thereby establishing its tissue-specific expression in *C. elegans*.

Genome wide analyses of microRNA expression in *C. elegans* using transgenes containing the putative promoters of microRNAs fused to the gene encoding green fluorescent protein have been reported previously [8,21]. *mir-58* was inferred to be expressed in the intestine, hypodermis, pharynx, spermatheca, excretory canal, and excretory cell soma [8]. Expression of *mir-58* was not observed in the nervous system. *mir-80* was shown to be expressed in the posterior intestine, head and body wall muscle, uterus, vulva, distal tip cells, excretory cells, dorsal nerve cord, and amphid neurons; *mir-81* was shown to be expressed weakly in head neurons; and *mir-82* was observed to be expressed in pharyngeal muscle, spermatheca, and a subset of both the ventral nerve cord and the amphid neurons [21]. The tissues in which these microRNAs are reportedly present are consistent with the absence of PMK-2 expression we observe in these tissues when miR-58/80-82 regulation is intact (Fig. 2B). Additionally, these data are consistent with the tissues that misexpress PMK-2 when miR-58/80-82 regulation is disabled (Fig. 2C-2D). Taken together with our data, these reported expression data on the miR-58/80-82 family suggest that the tissue-specific expression of PMK-2 is a consequence of distinct tissue-expression patterns of the corresponding microRNAs that target *pmk-2*.

MicroRNAs have been implicated to function both as a backup to reinforce transcriptional gene programs and as an instructive signal to shape gene expression patterns [22]. Expression analyses of microRNAs and their putative target transcripts in *Drosophila* and mammals showed largely nonoverlapping expression patterns, suggesting that microRNAs function secondarily to transcriptional mechanisms to reinforce promoter-defined spatial expression patterns of their targets at the post-transcriptional level [23–25]. Studies in zebrafish revealed overlapping expression patterns and observed that levels of transcripts are often lower in cells expressing the microRNA, leading to the hypothesis that microRNAs may also play a more active role in conjunction with transcriptional mechanisms to shape gene expression patterns of their targets [26]. Supporting this hypothesis is the genetic analysis of muscle microRNAs miR-1 and miR-133 in zebrafish, which were shown to refine the expression levels of their target transcripts in muscle [27]. We show nonoverlapping expression of PMK-2 and miR-58/80-82, established not by the promoter of these genes, but rather complete destabilization of *pmk-2* mRNA by miR-58/80-82, suggesting that the activity of microRNAs can define specific patterns of gene expression in different cell types.

Promoter and enhancer elements commonly direct tissue-specific gene expression in animals, and thus a role for miR-58 family-mediated post-transcriptional regulation in establishing the tissue expression pattern of *pmk-2* might be somewhat unexpected. For *pmk-2*, microRNA-mediated regulation may in part be a consequence of the co-transcription of both

pmk-2 and *pmk-1* from the same operon. Genes co-transcribed in eukaryotic operons need not share a similarity in function and are generally not thought to be expressed in specific tissues [28]. Our data suggest that microRNA-mediated regulation may contribute to differential tissue expression patterns of genes co-transcribed from the same operon in *C. elegans*.

MicroRNAs have been shown to establish cell fate through specific cell type expression during *C. elegans* development [29–31]. For example, the *lsy-6* microRNA regulates the development of left/right asymmetry through the specific repression of its target in one of two ASE chemosensory neurons [29]. In contrast to the highly selective expression and function of the *lsy-6* microRNA in a pair of chemosensory neurons, the miR-58 family functions in a large number of cells and tissues to restrict expression of PMK-2 in non-neuronal tissues at all developmental stages.

The systematic identification of microRNAs of *C. elegans* by deep sequencing determined that miR-58 is the most abundant microRNA, accounting for nearly half of all microRNAs present in *C. elegans* at all developmental stages [7]. The abundance of miR-58, along with the presence of multiple miR-58 binding sites in the 3'UTR of *pmk-2*, may contribute to the large increase of *pmk-2* mRNA and protein levels when miR-58 family regulation is inhibited. The approximately six-fold difference we observe in *pmk-2* mRNA levels and comparable difference in PMK-2 protein levels reflect quantitation from whole worm lysates in which PMK-2 is detectably expressed in the nervous system. Considering this and the lack of PMK-2 protein observed in non-neuronal tissues when miR-58 targeting is intact (i.e. wild type conditions), the magnitude of PMK-2 repression in non-neuronal tissues is likely much greater than six-fold. The switch-like "off" state of PMK-2 expression imposed by the miR-58/80-82 family in non-neuronal tissues in regulating the spatial expression of PMK-2 is reminiscent of the magnitude of target repression exhibited by *lin-4* and *let-7* microRNAs in the temporal control of developmental timing [2,32]. In addition, our data corroborate prior phenotypic analysis suggestive of redundancy among members of the miR-58 family [5,6], demonstrating redundant roles for miR-58, miR-80, miR-81, and miR-82 in the destabilization of *pmk-2* mRNA and corresponding repression of activated PMK-2 protein levels.

The broad and constitutive expression of *mir-58/80-82* suggests a more general housekeeping role for this microRNA family in the establishment and maintenance of tissue-specific gene expression by repressing the expression of neuronal-specific genes in non-neuronal tissues. Supporting this hypothesis is the genome-wide analysis of tissue-specific gene expression in *C. elegans*, which revealed enrichment for miR-58 binding sites among neuronal genes [33]. We speculate that the defects in size, locomotion, and egg-laying behavior observed in the *mir-58; mir-80; mir-81-82* mutant are due to the cumulative misexpression of miR-58/80-82 target genes in non-neuronal tissues, as *pmk-2* loss-of-function alone cannot suppress these defects (D.J.P. and D.H.K., unpublished observations).

The characterization of microRNAs expressed in specific tissues of mice revealed the presence of a single microRNA and/or microRNA family in high abundance [34], raising the possibility that such microRNAs might function to establish tissue expression patterns. Our data on the regulation of PMK-2 tissue expression by the miR-58 family provide genetic evidence for this hypothesis and point to a more general role for the highly abundant miR-58 family in the maintenance of tissue-specific gene expression.

Materials and Methods

Strains

All *C. elegans* strains were maintained and propagated on *E. coli* OP50 as described previously [35]. N2 was the wild-type strain. The following mutations were used in this study:

LGI: *kyIs140[str-2::GFP, lin-15(+)]*

LGIII: *agIs219[$P_{T24B8.5}$::GFP, $P_{ttx\text{-}3}$::GFP], tir-1(qd4), mir-80(nDf53)*

LGIV: *mir-58(n4640), pmk-2(qd284), pmk-2(qd307), pmk-2(qd287), pmk-2(qd279), pmk-2(qd280), pmk-2(qd305), pmk-2(qd171), pmk-1(km25)*

LGX: *mir-81-82(nDf54), sek-1(km4), nIs145[$P_{tph\text{-}1}$::GFP, lin-15(+)]*

Extrachromosomal arrays: *qdEx101[P_{operon}::islo-1::pmk-3::pmk-2::GFP::pmk-1::mCherry], qdEx102[P_{operon}::islo-1::pmk-3::pmk-2$_{mut}$::GFP::pmk-1::mCherry]*

A list of all strains used in this study is provided in the Supporting Information (S1 Table).

Pathogenesis assays

Cultures of *P. aeruginosa* PA14 were grown in Luria-Bertani (LB) broth overnight at 37°C. Five microliters of the overnight culture was used to seed 35-mm slow-kill assay plates (1.7% agar, 0.35% peptone, 0.3% sodium chloride, 5 μg/ml cholesterol, 1 mM calcium chloride, 1 mM magnesium sulfate, 25 mM potassium phosphate) containing 50 μg/ml 5-fluorodeoxyuridine (FUdR), used to prevent eggs from hatching. The culture was seeded in the middle of the plates and was not spread to the edges, meaning the resulting lawn would be "small," allowing for behavioral avoidance. The seeded plates were incubated overnight at 37°C and then overnight at room temperature. Roughly 40 L4 larval stage worms were placed onto a plate containing the prepared *P. aeruginosa* bacterial lawn with four plates per strain. The assay was carried out at 25°C. Plates were checked at the indicated times and worms that did not respond to a gentle prod from a platinum wire were scored as dead. Worms that crawled off of the plate or burrowed were censored.

$P_{tph\text{-}1}$::*GFP* imaging and quantification of GFP fluorescence

Adult worms (specifically, 12–16 hr post L4 larval stage at 25°C) were transferred to plates containing either a lawn of non-pathogenic *E. coli* OP50 or pathogenic *P. aeruginosa* PA14 and incubated at 25°C for 6 hr at which time the worms were immobilized with 50 mM sodium azide and mounted on 2% agarose pads. Immobilized worms were viewed using an AxioImager Z1 fluorescence microscope (Carl Zeiss AG, Oberkochen, Germany) with an EC Plan-Neofluar 40x/1.3 Oil DIC objective and the focal plane with the strongest GFP signal in the ADF neuron was used to take a 30 ms exposure picture with an AxioCam HRm camera. The images were analyzed in Fiji [36], where the ADF neuron was located and the pixel intensity values were examined. The maximum pixel intensity value in the ADF neuron was used as the $P_{tph\text{-}1}$::*GFP* fluorescence value for each worm. In each experiment, 7–10 worms of the indicated genotype were imaged for each condition (OP50, PA14). For the experiment shown in Fig. 1E, one outlier was identified (in the *tir-1(qd4)* mutant, *P. aeruginosa* exposure dataset) using Grubbs' test and excluded from the graph and analysis. Statistical analyses of changes in fluorescence were performed in Prism 5 (GraphPad Software, Inc., La Jolla, CA) using a two-way ANOVA and Bonferroni post-test.

Isolation of *pmk-2(qd284)*, *pmk-2(qd287)*, and *pmk-2(qd307)*

The *qd284*, *qd287*, and *qd307* alleles of *pmk-2* were isolated by CRISPR-Cas9-mediated genome editing as described previously [37]. Two separate *pmk-2* single guide RNA (sgRNA) expression vectors derived from pUC57 were constructed following the published protocol. Both sgRNAs target sequences located in what corresponds to the first exon of the *pmk-2* transcript. The target sequences were chosen to contain restriction enzyme recognition sites to facilitate the screening for mutations. Germline transformation was performed as described previously [38] using the following plasmids: 50 ng/μl $P_{eft\text{-}3}$::*Cas9-SV40 NLS*::*tbb-2 3'UTR*, 45 ng/μl

P_{U6}:: *pmk-2 sgRNA*, 5 ng/μl pCFJ104[P_{myo-3}::*mCherry*]. Screening for mutations was performed as outlined [37]. To confirm that a frameshift in the first exon of *pmk-2* results in a null allele, the *qd284* mutation was crossed into the *mir-80; mir-58; mir-81-82* mutant and levels of activated PMK-2 protein were determined. Activated PMK-2 protein was not detected in the *mir-80; mir-58 pmk-2(qd284); mir-81-82* mutant, indicating that these mutations are null (S2 Fig.).

Isolation of *pmk-2(qd171)*

The *qd171* allele of *pmk-2* was isolated from a screen for suppressors of the enhanced susceptibility to pathogen (Esp) phenotype conferred by the *km25* loss-of-function allele of *pmk-1*. *pmk-1(km25)* mutant L4 larvae carrying the *agIs219* reporter transgene for p38 activity in the intestine were mutagenized with ethyl methanesulfonate (EMS) as described previously [39]. Synchronized F_2 progeny from roughly 35,000 mutagenized genomes were screened for an increase in expression of GFP from the *agIs219* reporter transgene using a dissecting microscope equipped to detect GFP fluorescence. Mutants with increased expression of GFP were tested individually for the ability to suppress the Esp phenotype conferred by loss of *pmk-1* function. Mutants that suppressed the Esp phenotype conferred by loss of *pmk-1* function were then subsequently screened for suppression of the diminished expression of *pmk-1* target genes conferred by loss of *pmk-1* function. Single-nucleotide polymorphism (SNP)-based mapping using the *C. elegans* isolate CB4856 was performed as described previously [40,41]. One of the suppressors from this screen, *qd171*, mapped to a region on LGIV containing the *pmk* operon. Sequence determination of *pmk-1* and *pmk-2* revealed that *qd171* was an allele of *pmk-2*.

Isolation of *pmk-2(qd305)*

The *qd305* deletion allele of *pmk-2* was isolated by CRISPR-Cas9-mediated genome editing as described previously [37]. Three separate *pmk-2* sgRNA expression vectors derived from pUC57 were constructed following the published protocol. All three sgRNAs target sequences located within or directly downstream of the 3'UTR of *pmk-2*. Germline transformation was performed as described previously [38] using the following plasmids: 50 ng/μl P_{eft-3}::*Cas9-SV40 NLS*::*tbb-2 3'UTR*, 50 ng/μl of each of the three P_{U6}::*pmk-2 3'UTR sgRNA*, 5 ng/μl pCFJ104 [P_{myo-3}::*mCherry*]. Screening for deletions was performed by PCR analysis of F_1 transgenic animals.

Isolation of *pmk-2(qd279 qd171)* and *pmk-2(qd280 qd171)*

The *qd279* and *qd280* alleles of *pmk-2* were isolated from a screen for suppressors of the *pmk-2 (qd171)* suppressor phenotype of the *pmk-1(km25)* loss-of-function phenotype. *pmk-2(qd171) pmk-1(km25)* mutant L4 larvae carrying the *agIs219* reporter transgene for p38 activity in the intestine were mutagenized with EMS as described previously [39]. Synchronized F_2 progeny from roughly 14,000 mutagenized genomes were screened for a decrease in expression of GFP from the *agIs219* reporter transgene using a dissecting microscope equipped to detect GFP fluorescence. The sequence of *pmk-2* was determined in mutants with diminished GFP expression from the *agIs219* transgene. We identified three alleles of *pmk-2*: *qd279* and *qd280* (which are missense mutations in conserved residues and were utilized in this study), as well as *qd281*.

RNA isolation, 3' RACE, and quantitative RT-PCR

Hypochlorite-synchronized populations of L4 larval stage worms were flash-frozen in liquid nitrogen and stored at -80°C until RNA extraction using TRI reagent (Ambion, Life Technologies, Thermo Fisher Scientific, Inc., Waltham, MA). Strain MT15563, carrying mutations in

mir-58,80-82, grew slower than the other strains and therefore was harvested ~10 hr after the wild type strain. For 3' RACE experiments, cDNA was prepared using the FirstChoice RLM-RACE Kit (Ambion). Two successive rounds of PCR were performed using nested primers specific to *pmk-2* and an adaptor at the 3'-end. Sequence determination was performed by direct Sanger sequencing of 3' RACE products. miR-58/80-82 seed match sites were identified using TargetScan [42]. For quantitative RT-PCR experiments, cDNA was prepared with the RETROscript Kit (Ambion) using oligo dT primers. qRT-PCR was performed with a Mastercycler Realplex (Eppendorf AG, Hamburg, Germany) with SYBR Green detection (Roche Diagnostics Corp., Indianapolis, IN) in triplicate 20 μl reactions. *pmk-1*, *pmk-2*, and *pmk-3* mRNA levels were normalized to the control gene *snb-1*. Fold change relative to wild type was determined using the Pfaffl method [43]. Sequences of primers used for qRT-PCR: *pmk-1*, tgaatgatgatgtaagggcaga and cttcctcttcgtcagcaaatg; *pmk-2*, caagtgttacgtgggctcaa and cgagaatcttgacttcgcatc; *pmk-3*, gtatcgaagcaacgggaaac and tggaccacatggttttgaga; *snb-1*, ccggataagaccatcttgacg and gacgacttcatcaacctgagc.

Immunoblotting

For the experiments with *pmk-1(km25)*, *pmk-2(qd171) pmk-1(km25)*, and *pmk-2(qd279 qd171) pmk-1(km25)* strains with RNAi treatment (Fig. 3D), mixed stage populations of worms subjected to RNAi for multiple generations were used for Western analysis. For experiments with *mir-58/80-82* family microRNA deletion strains (Fig. 4C and S2 Fig.) and *pmk-2(qd305)* (S1C Fig.), hypochlorite-synchronized populations of L4 larval worms were used for Western analysis. Strain MT15563, carrying mutations in *mir-58,80-82*, grew slower than the other strains and therefore was harvested ~10 hr after the wild type strain. Worms were collected, washed twice with M9, incubated in M9 while rotating at 20°C to clear the gut of bacteria, washed again twice with M9, and then pelleted. An equal volume of 2x lysis buffer (4% SDS, 100 mM Tris HCl pH 6.8, and 20% glycerol) was added to the worm pellets, which were then boiled for 15 minutes with occasional vortexing and then centrifuged to pellet the debris. The protein concentration of the lysates (supernatant from the previous step) was determined using the BCA Protein Assay Kit (Pierce, Thermo Fisher Scientific, Inc., Waltham, MA). For each sample, 50 μg of total protein was separated on a 10% SDS-PAGE gel (Bio-Rad Laboratories, Inc., Hercules, CA) and then transferred to a nitrocellulose membrane (GE Healthcare, Little Chalfont, United Kingdom). Blots were blocked with TBST supplemented with 5% skim milk power and then probed with either a 1:1,000 dilution of rabbit anti-ACTIVE p38 MAPK pAb (Promega Corp., Madison, WI), which recognizes the dually phosphorylated TGY motif of activated p38 MAPK, or a 1:10,000 dilution of mouse anti-β-tubulin (E7 Developmental Hybridoma Bank, Iowa City, IA) in TBST with 5% skim milk powder. Horseradish peroxidase (HRP)-conjugated anti-rabbit and anti-mouse IgG secondary antibodies (Cell Signaling Technology, Inc., Danvers, MA) were used followed by detection with ECL reagents (GE Healthcare).

RNA interference

Feeding RNAi was performed as previously described [44]. NGM agar plates supplemented with 2 mM isopropyl-β-D-thiogalactopyranoside (IPTG) and 25 μg/ml of carbenicillin were seeded with *E. coli* HT115 bacterial cultures carrying the control plasmid pPD129.36 (Ligation number L4440) or carrying specific plasmids derived from pPD129.36 designed to target either *sek-1* or *pmk-2* for RNAi. For pathogenesis assays and visualization of GFP expression from the *agIs219* reporter, 3–6 L4 larval stage worms were placed onto seeded RNAi plates and their

progeny were assayed. For Western analysis, ~10 L4 larval stage worms were placed onto seeded RNAi plates and the progeny of these worms were harvested before being deprived of food.

Plasmids

The *pmk* operon translational reporter was constructed through ligation of overlapping PCR amplicons by homologous recombination in *Saccharomyces cerevisiae* strain FY2 as previously described [45]. A ~20.3 kb region containing the *pmk* operon and ~4.4 kb of upstream sequence was amplified from fosmid 34bC01 in adjacent fragments with at least 50 bp of overlap between fragments. For the *pmk* operon translational reporter carrying mutations in the 3'UTR of *pmk-2*, 90 bp reverse complement primers containing the desired mutations were designed and used to amplify the 3'UTR of *pmk-2*. The gene encoding GFP was amplified from pPD95.75 (Addgene, Cambridge, MA). The gene encoding mCherry was amplified from pCFJ90 (Addgene). The PCR amplicons were transformed into *S. cerevisiae* strain FY2 along with destination vector pNP30 (gift of N. Paquin, pNP30 is a pRS426-derived plasmid compatible with MosSCI integration at locus *ttTi5605* on LGII) digested with XhoI and AvrII (New England Biosciences, Inc., Ipswich, MA). Yeast DNA was extracted with phenol-chloroform and transformed into DH5-α electrocompetent cells (Protein Express, Inc., Cincinnati, OH). Plasmids were prepped using a Miniprep Kit (Qiagen N.V., Venlo, Netherlands) and their sequences were verified. Germline transformations were performed as described previously [38].

Microscopy

To visualize expression of GFP from the *agIs219* reporter transgene, adult worms (16–24 hr post L4 larval stage at 20°C) were picked over to an unseeded NGM agar plate and immobilized with 50 mM sodium azide. Worms were viewed using a Stereo V12 fluorescence microscope (Zeiss) and pictures were taken with an AxioCam MRc camera. To visualize expression of GFP and mCherry from the *pmk* operon translational reporter, L4 larval stage transgenic animals were immobilized with 50 mM sodium azide and mounted on 2% agarose pads. Worms were viewed using a LSM510 confocal microscope (Zeiss). Stacks of confocal images were acquired and processed in Fiji to obtain maximum projections [36]. All images were prepared in Photoshop (Adobe Systems, Inc., San Jose, CA).

Supporting Information

S1 Fig. An independent deletion in the 3'UTR of *pmk-2*, *qd305*, confers an increase in levels of *pmk-2* mRNA and activated protein. (A) DNA sequence of the *pmk-2* 3'UTR (release WS245). Uppercase, last exon of *pmk-2* and first exon of *pmk-1*, respectively. Underline, polyadenylation signal. Bold font, miR-58/80-82 family seed match site. Gray highlight, *qd305* deletion. (B) qRT-PCR analysis of *pmk-1*, *pmk-2*, and *pmk-3* mRNA levels in L4 larval stage wild type worms and the *pmk-2(qd305)* mutant. Levels of *pmk-1*, *pmk-2*, and *pmk-3* mRNA are normalized to the levels of *snb-1* mRNA. Values plotted are the fold changes relative to wild type. Shown is the mean ± SEM (n = 3 independent biological replicates, $***$ $P<0.001$, two-way ANOVA with Bonferroni post-test). (C) Immunoblot analysis of lysates from L4 larval stage wild type worms, *pmk-2(qd305)* mutant animals, and *pmk-2(qd307 qd305)* intragenic suppressor mutant animals using antibodies that recognize activated p38 MAPK and β-tubulin. (PDF)

S2 Fig. The *qd284* allele of *pmk-2* does not produce activated PMK-2 protein. Immunoblot analysis of lysates from L4 larval stage wild type worms, *mir-80; mir-58; mir-81-82* mutant animals, and *mir-80; mir-58 pmk-2(qd284); mir-81-82* mutant animals using antibodies that

recognize activated p38 MAPK and β-tubulin.
(PDF)

S1 Table. Strains used in this study.
(PDF)

Acknowledgments

We thank N. An, H. R. Horvitz, and N. Paquin for strains and reagents. We thank N. Bhatla, K. Boulias, C. Burge, S. Fischer, and N. Paquin for discussions.

Author Contributions

Conceived and designed the experiments: DJP DHK. Performed the experiments: DJP ERK. Analyzed the data: DJP DHK. Wrote the paper: DJP DHK.

References

1. Lee RC, Feinbaum RL, Ambros V (1993) The C. elegans heterochronic gene lin-4 encodes small RNAs with antisense complementarity to lin-14. Cell 75: 843–854. PMID: 8252621
2. Reinhart BJ, Slack FJ, Basson M, Pasquinelli AE, Bettinger JC, et al. (2000) The 21-nucleotide let-7 RNA regulates developmental timing in Caenorhabditis elegans. Nature 403: 901–906. PMID: 10706289
3. Lau NC, Lim LP, Weinstein EG, Bartel DP (2001) An Abundant Class of Tiny RNAs with Probable Regulatory Roles in Caenorhabditis elegans. Science 294: 858–862. PMID: 11679671
4. Lee RC, Ambros V (2001) An Extensive Class of Small RNAs in Caenorhabditis elegans. Science 294: 862–864. PMID: 11679672
5. Miska EA, Alvarez-Saavedra E, Abbott AL, Lau NC, Hellman AB, et al. (2007) Most Caenorhabditis elegans microRNAs are individually not essential for development or viability. PLoS Genet 3: e215. PMID: 18085825
6. Alvarez-Saavedra E, Horvitz HR (2010) Many Families of C. elegans MicroRNAs Are Not Essential for Development or Viability. Curr Biol 20: 367–373. doi: 10.1016/j.cub.2009.12.051 PMID: 20096582
7. Kato M, de Lencastre A, Pincus Z, Slack FJ (2009) Dynamic expression of small non-coding RNAs, including novel microRNAs and piRNAs/21U-RNAs, during Caenorhabditis elegans development. Genome Biol 10: R54. doi: 10.1186/gb-2009-10-5-r54 PMID: 19460142
8. Isik M, Korswagen HC, Berezikov E (2010) Expression patterns of intronic microRNAs in Caenorhabditis elegans. Silence 1: 5. doi: 10.1186/1758-907X-1-5 PMID: 20226079
9. Jovanovic M, Reiter L, Clark A, Weiss M, Picotti P, et al. (2012) RIP-chip-SRM—a new combinatorial large-scale approach identifies a set of translationally regulated bantam/miR-58 targets in C. elegans. Genome Res 22: 1360–1371. doi: 10.1101/gr.133330.111 PMID: 22454234
10. Berman K, McKay J, Avery L, Cobb M (2001) Isolation and characterization of pmk-(1–3): three p38 homologs in Caenorhabditis elegans. Mol Cell Biol Res Commun MCBRC 4: 337–344. PMID: 11703092
11. Kim DH, Feinbaum R, Alloing G, Emerson FE, Garsin DA, et al. (2002) A conserved p38 MAP kinase pathway in Caenorhabditis elegans innate immunity. Science 297: 623–626. PMID: 12142542
12. Shivers RP, Kooistra T, Chu SW, Pagano DJ, Kim DH (2009) Tissue-specific activities of an immune signaling module regulate physiological responses to pathogenic and nutritional bacteria in C. elegans. Cell Host Microbe 6: 321–330. doi: 10.1016/j.chom.2009.09.001 PMID: 19837372
13. Liberati NT, Fitzgerald KA, Kim DH, Feinbaum R, Golenbock DT, et al. (2004) Requirement for a conserved Toll/interleukin-1 resistance domain protein in the Caenorhabditis elegans immune response. Proc Natl Acad Sci U S A 101: 6593–6598. PMID: 15123841
14. Couillault C, Pujol N, Reboul J, Sabatier L, Guichou J-F, et al. (2004) TLR-independent control of innate immunity in Caenorhabditis elegans by the TIR domain adaptor protein TIR-1, an ortholog of human SARM. Nat Immunol 5: 488–494. PMID: 15048112
15. Chuang C-F, Bargmann CI (2005) A Toll-interleukin 1 repeat protein at the synapse specifies asymmetric odorant receptor expression via ASK1 MAPKKK signaling. Genes Dev 19: 270–281. PMID: 15625192

16. Tanaka-Hino M, Sagasti A, Hisamoto N, Kawasaki M, Nakano S, et al. (2002) SEK-1 MAPKK mediates Ca2+ signaling to determine neuronal asymmetric development in Caenorhabditis elegans. EMBO Rep 3: 56–62. PMID: 11751572

17. Sagasti A, Hisamoto N, Hyodo J, Tanaka-Hino M, Matsumoto K, et al. (2001) The CaMKII UNC-43 activates the MAPKKK NSY-1 to execute a lateral signaling decision required for asymmetric olfactory neuron fates. Cell 105: 221–232. PMID: 11336672

18. Zhang Y, Lu H, Bargmann CI (2005) Pathogenic bacteria induce aversive olfactory learning in Caenorhabditis elegans. Nature 438: 179–184. PMID: 16281027

19. Shivers RP, Pagano DJ, Kooistra T, Richardson CE, Reddy KC, et al. (2010) Phosphorylation of the conserved transcription factor ATF-7 by PMK-1 p38 MAPK regulates innate immunity in Caenorhabditis elegans. PLoS Genet 6: e1000892. doi: 10.1371/journal.pgen.1000892 PMID: 20369020

20. Lewis BP, Burge CB, Bartel DP (2005) Conserved seed pairing, often flanked by adenosines, indicates that thousands of human genes are microRNA targets. Cell 120: 15–20. PMID: 15652477

21. Martinez NJ, Ow MC, Reece-Hoyes JS, Barrasa MI, Ambros VR, et al. (2008) Genome-scale spatiotemporal analysis of Caenorhabditis elegans microRNA promoter activity. Genome Res 18: 2005–2015. doi: 10.1101/gr.083055.108 PMID: 18981266

22. Ebert MS, Sharp PA (2012) Roles for MicroRNAs in Conferring Robustness to Biological Processes. Cell 149: 515–524. doi: 10.1016/j.cell.2012.04.005 PMID: 22541426

23. Stark A, Brennecke J, Bushati N, Russell RB, Cohen SM (2005) Animal MicroRNAs Confer Robustness to Gene Expression and Have a Significant Impact on 3′UTR Evolution. Cell 123: 1133–1146. PMID: 16337999

24. Farh KK-H, Grimson A, Jan C, Lewis BP, Johnston WK, et al. (2005) The Widespread Impact of Mammalian MicroRNAs on mRNA Repression and Evolution. Science 310: 1817–1821. PMID: 16308420

25. Sood P, Krek A, Zavolan M, Macino G, Rajewsky N (2006) Cell-type-specific signatures of microRNAs on target mRNA expression. Proc Natl Acad Sci U S A 103: 2746–2751. PMID: 16477010

26. Shkumatava A, Stark A, Sive H, Bartel DP (2009) Coherent but overlapping expression of microRNAs and their targets during vertebrate development. Genes Dev 23: 466–481. doi: 10.1101/gad.1745709 PMID: 19240133

27. Mishima Y, Abreu-Goodger C, Staton AA, Stahlhut C, Shou C, et al. (2009) Zebrafish miR-1 and miR-133 shape muscle gene expression and regulate sarcomeric actin organization. Genes Dev 23: 619–632. doi: 10.1101/gad.1760209 PMID: 19240126

28. Blumenthal T (2012) Trans-splicing and operons in C. elegans. WormBook Online Rev C Elegans Biol: 1–11.

29. Johnston RJ, Hobert O (2003) A microRNA controlling left/right neuronal asymmetry in Caenorhabditis elegans. Nature 426: 845–849. PMID: 14685240

30. Chang S, Johnston RJ, Frøkjaer-Jensen C, Lockery S, Hobert O (2004) MicroRNAs act sequentially and asymmetrically to control chemosensory laterality in the nematode. Nature 430: 785–789. PMID: 15306811

31. Yoo AS, Greenwald I (2005) LIN-12/Notch Activation Leads to MicroRNA-Mediated Down-Regulation of Vav in C. elegans. Science 310: 1330–1333. PMID: 16239437

32. Lee RC, Feinbaum RL, Ambros V (1993) The C. elegans heterochronic gene lin-4 encodes small RNAs with antisense complementarity to lin-14. Cell 75: 843–854. PMID: 8252621

33. Spencer WC, Zeller G, Watson JD, Henz SR, Watkins KL, et al. (2011) A spatial and temporal map of C. elegans gene expression. Genome Res 21: 325–341. doi: 10.1101/gr.114595.110 PMID: 21177967

34. Lagos-Quintana M, Rauhut R, Yalcin A, Meyer J, Lendeckel W, et al. (2002) Identification of tissue-specific microRNAs from mouse. Curr Biol CB 12: 735–739.

35. Brenner S (1974) The genetics of Caenorhabditis elegans. Genetics 77: 71–94. PMID: 4366476

36. Schindelin J, Arganda-Carreras I, Frise E, Kaynig V, Longair M, et al. (2012) Fiji: an open-source platform for biological-image analysis. Nat Methods 9: 676–682. doi: 10.1038/nmeth.2019 PMID: 22743772

37. Friedland AE, Tzur YB, Esvelt KM, Colaiácovo MP, Church GM, et al. (2013) Heritable genome editing in C. elegans via a CRISPR-Cas9 system. Nat Methods 10: 741–743. doi: 10.1038/nmeth.2532 PMID: 23817069

38. Mello CC, Kramer JM, Stinchcomb D, Ambros V (1991) Efficient gene transfer in C.elegans: extrachromosomal maintenance and integration of transforming sequences. EMBO J 10: 3959–3970. PMID: 1935914

39. Jorgensen EM, Mango SE (2002) The art and design of genetic screens: caenorhabditis elegans. Nat Rev Genet 3: 356–369. PMID: 11988761

40. Wicks SR, Yeh RT, Gish WR, Waterston RH, Plasterk RH (2001) Rapid gene mapping in Caenorhabditis elegans using a high density polymorphism map. Nat Genet 28: 160–164. PMID: 11381264

41. Davis MW, Hammarlund M, Harrach T, Hullett P, Olsen S, et al. (2005) Rapid single nucleotide polymorphism mapping in C. elegans. BMC Genomics 6: 118. PMID: 16156901

42. Friedman RC, Farh KK-H, Burge CB, Bartel DP (2009) Most mammalian mRNAs are conserved targets of microRNAs. Genome Res 19: 92–105. doi: 10.1101/gr.082701.108 PMID: 18955434

43. Pfaffl MW (2001) A new mathematical model for relative quantification in real-time RT-PCR. Nucleic Acids Res 29: e45. PMID: 11328886

44. Fraser AG, Kamath RS, Zipperlen P, Martinez-Campos M, Sohrmann M, et al. (2000) Functional genomic analysis of C. elegans chromosome I by systematic RNA interference. Nature 408: 325–330. PMID: 11099033

45. Andersen EC (2011) PCR-directed in vivo plasmid construction using homologous recombination in baker's yeast. Methods Mol Biol Clifton NJ 772: 409–421. doi: 10.1007/978-1-61779-228-1_24 PMID: 22065452

46. Siepel A, Bejerano G, Pedersen JS, Hinrichs AS, Hou M, et al. (2005) Evolutionarily conserved elements in vertebrate, insect, worm, and yeast genomes. Genome Res 15: 1034–1050. PMID: 16024819
